# Effects of a neuromuscular training program using external focus attention cues in male athletes with anterior cruciate ligament reconstruction: a randomized clinical trial

**DOI:** 10.1186/s13102-021-00275-3

**Published:** 2021-05-08

**Authors:** Mohamad Ghaderi, Amir Letafatkar, Abbey C. Thomas, Sohrab Keyhani

**Affiliations:** 1 Faculty of Physical Education and Sports Sciences, Kharazmi University, Tehran, Iran; 2 Sport Injury and Corrective Exercises, Kharazmi University, Tehran, Iran; 3 Biomechanics and Corrective Exercise Laboratory, Faculty of Physical Education and Sport sciences, Kharazmi University, Mirdamad Blvd., Hesari St, Tehran, Iran; 4 Department of Kinesiology, University of North Carolina at Charlotte, Charlotte, NC USA; 5 Orthopedic Department Chair, Akhtar Orthopedic Hospital, Shahid Beheshti University of Medical Sciences, Tehran, Iran

**Keywords:** Anterior cruciate ligament reconstruction, Neuromuscular training, Rehabilitation, External focus attention

## Abstract

**Background:**

Athletes who have undergone anterior cruciate ligament (ACL) reconstruction often exhibit persistent altered biomechanics and impaired function. Neuromuscular training programs appear to be effective for reducing high-risk landing mechanics and preventing primary ACL injuries; however, there have been few attempts to examine their effects in athletes who have undergone ACL reconstruction. The purpose of our study was to examine the effects of a neuromuscular training program that emphasizes external focus of attention cuing on biomechanics, knee proprioception, and patient-reported function in athletes who had undergone ACL reconstruction and completed conventional post-operative rehabilitation.

**Methods:**

Twenty-four male athletes who had undergone primary, unilateral, hamstring autograft ACL reconstruction and completed conventional post-operative rehabilitation were randomly allocated to an experimental group (*n* = 12) who took part in an 8-week neuromuscular training program or a control group (*n* = 12) who continued a placebo program. The neuromuscular training program included lower extremity strengthening and plyometric exercises, balance training, and movement pattern re-training. Biomechanics during single-leg landing, knee proprioception, and patient-reported function were assessed before and after the 8-week training period.

**Results:**

Athletes in the experimental group demonstrated increased trunk, hip, and knee flexion angles and decreased knee abduction, internal rotation angles and knee valgus during landing following the intervention. Further, the experimental group decreased their peak knee extension and abduction moments and vertical ground reaction force on landing post-intervention. International Knee Documentation Committee questionnaire (IKDC) scores increased in the experimental group following training. The control group demonstrated no changes in any variable over the same time period.

**Conclusions:**

Neuromuscular training with external focus of attention cueing improved landing biomechanics in patients after ACL reconstruction. Neuromuscular training programs beneficially mitigate second ACL injury risk factors and should be emphasized during and after traditional post-operative rehabilitation.

**Trial registration:**

Current Controlled Trials using the IRCT website with ID number of, IRCT20180412039278N1 “Prospectively registered” at 21/12/2018.

## Background

Anterior cruciate ligament (ACL) injuries mostly occur during sports activities that include sudden stops, changes in direction, jumping, or landing [1]. Immediately after this injury, the athlete is confronted with multi-planar biomechanical asymmetries, loss of a season in their respective sport, a long, difficult recovery ahead, and a possible reduction in performance following a return to sport (RTS) [1, 2]. Nearly two-thirds of athletes do not return to preinjury level one year after ACL reconstruction [3]. In fact, only 65% of athletes return to the pre-injury level of sport at a mean follow-up of nearly 3.5 years despite recovering normal knee function [4]. Also, seven years after ACL reconstruction, only 36% still participated in their original sports [5]. Moreover, for those who do resume their previous level of activity, the risk of a second ACL injury to the ipsilateral or the contralateral knee due to reduced muscle strength and function may be as high as 29% [6].

Aberrant jump-landing biomechanics, particularly increased vertical ground reaction force (vGRF), decreased hip and knee flexion, and increased knee abduction and internal rotation, which collectively lead to dynamic “knee valgus”, have been associated with second ACL injury risk [7, 8]. These biomechanical components of second ACL injury risk may be effectively addressed with targeted neuromuscular training prior to unrestricted sports participation [9].

Neuromuscular training programs, which incorporate lower extremity strengthening exercises, plyometric exercises, balance training, and movement pattern re-training are recommended for primary prevention of ACL injuries [9, 10]. Programs of this nature appear to reduce ACL injury rates [9] and promote safer landing mechanics in athletes without a history of ACL injury [11].

Neuromuscular training has been demonstrated to mitigate biomechanical risk factors associated with ACL injury. For example, neuromuscular training programs 1) with added feedback reduced knee valgus angles and moments [11]; 2) with verbal feedback on incorrect technique improved VGRF [12]; 3) neuromuscular improved the H/Q ratio in female athletes [11]; and 4) increased the activity of the medial hamstrings in the pre landing phase which is thought to be beneficial for stabilizing the knee [13]. Specific to patients after ACL reconstruction, neuromuscular training has been demonstrated to significantly improved knee pain and global knee function compared with the traditional strength training. The authors also emphasized including neuromuscular training in the rehabilitation program after ACL reconstruction [14]. In addition, Shim et al., (2015) reported another benefit of neuromuscular training is that it reduces the anterior tibial displacement of the affected knee joints during standing, which, in turn, reduces ACL strain. Finally, neuromuscular training evoked higher muscle activation of the vastus medialis oblique, vastus lateralis, biceps femoris, and semitendinosus, all of which may improve functional joint stability [15].

Since the potential mechanism underlying the secondary injury is multifactorial (age, surgical procedure, and post-injury activity level [16], more focus on these factors into post-operative rehabilitation seems warranted. However, modification of these programs to emphasize an external focus of attention may be necessary to reduce risk factors of second ACL injury. Instructions that promote an internal focus of attention, which is common in rehabilitation, direct an athlete to attend to a specific aspect of their movement, whereas instructions that promote an external focus direct an athlete to attend to the effects of their movement [17]. For example, when an athlete is performing a hop for distance, they could be instructed to focus on extending their knee as rapidly as possible (internal focus) or pushing against the ground as forcefully as possible (external focus). Although the difference in these instructions appears subtle, training with an external focus has been shown to result in better performance, retention, transfer, and greater movement automaticity for a wide range of movement tasks [17]. A key difference from a motor learning standpoint between internal and external focus of attention cueing is that external focus promotes automaticity of movement [17], meaning the individual is not constrained in their movement profile and are freer to adapt to a changing environment. In recent years, Gokeler et al., (2015) determined the effect of an internal vs. external attentional focus on single leg hop distance and knee kinematics in patients after ACL reconstruction and reported biomechanical outcomes’ improvements for the injured legs after receiving external focus of attention training [18]. They concluded that using an external focus during rehabilitation of patients after ACL reconstruction promotes safer movement patterns compared to an internal focus of attention; thus, external may reduce second ACL injury risk [18].

Therefore, the purpose of our study was to examine the effects of a neuromuscular training program that emphasizes external focus of attention cuing on biomechanics, knee proprioception, and patient-reported function in athletes who had undergone ACL reconstruction and completed conventional post-operative rehabilitation. We expected that athletes who participated in neuromuscular training would exhibit improvements in biomechanics, knee proprioception, and function that exceed those exhibited by athletes who simply continued their typical training routine.

## Methods

Twenty-four male athletes participated in this randomized controlled trial (RCT) that was prospectively registered at [IRCT20180412039278N1, date of first registration *21/12/2018*].

**Fig. 1:**
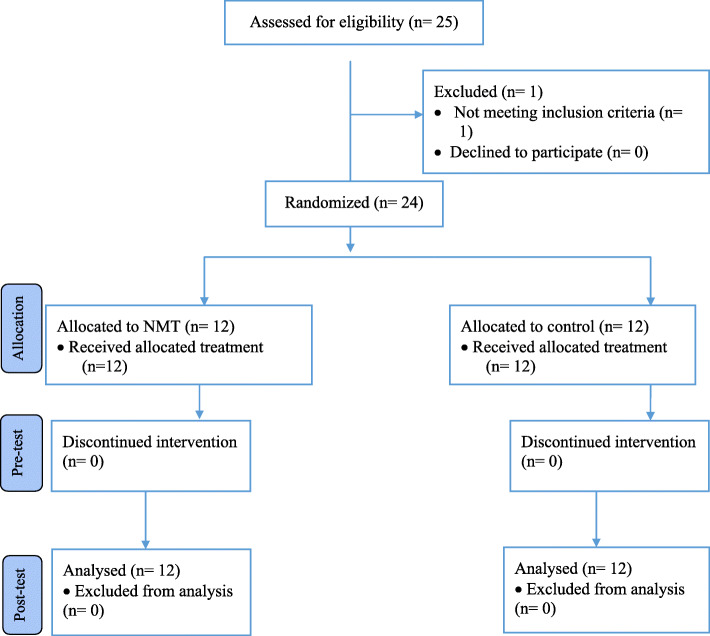
Consort Flow Diagram

A sample size estimate indicated that 12 participants per group (24 total athletes) would provide adequate statistical power to detect a group-by-time interaction for a moderate effect size (partial eta squared = 0.06) [19]. This determination was made based on biomechanical and joint position sense data. These data suggest joint position sense can change significantly following neuromuscular training, which yielded a large effect size [20]. Using these data, an alpha of 0.05, a beta of 0.20, the aforementioned moderate effect size of η^2^ = 0.06, and assuming a correlation among repeated measures of 0.85 for our sample size estimate, we arrived at the total 0f 24 participants needed. The value used for the correlation among repeated measures was based on the test-retest reliability reported for isokinetic testing [16]. G*Power software was used for sample size estimation [21] (Fig. 1).

Athletes were required to have undergone a successful primary, unilateral hamstring tendon autograft ACL reconstruction, performed by the same surgeon, and were cleared to resume sports participation by their medical team. All athletes intended to return to sports, such as soccer, that involve frequent landing and cutting. Clearance for return-to-sport was primarily based on the time since surgery, which is typical [22]. At the time of enrollment in the study, all athletes had undergone ACL reconstruction within the previous 6–12 months. Athletes who sustained a concomitant injury to another knee structure (e.g. medial collateral ligament, meniscus), had a history of previous musculoskeletal surgery to either leg, or experienced a post-operative re-injury were excluded from participating. The study protocol was approved by the Institutional Review Board at [omitted for review] and all participants provided written informed consent prior to enrollment.

Upon enrollment in the study, the first licensed athletic trainer conducted a preliminary assessment to ensure that it was safe for the athlete to participate in the activities associated with our study. This involved assessing knee pain, effusion, 80% quadriceps strength limb symmetry via handle-held dynamometer, and knee range of joint motion via electro goniometer, as well as observing single leg hopping (i.e., single leg forward hop, triple hop, crossover hop, and 6 m timed hop as previously described) [23]. Athletes were required to exhibit no effusion, report pain-free knee active range of motion, and complete all hop tests without pain and at an equivalent distance/rate of at least 80% of the contralateral limb. All athletes who enrolled in the study were deemed safe to participate. Athletes were randomly allocated to an experimental group (*n* = 12) or a control group (*n* = 12). Randomization was performed by an independent investigator not familiar with the testing protocol using a random number table. Group allocation was concealed by means of an opaque envelope until after athletes had been enrolled in the study to minimize potential bias. A baseline assessment of hamstrings and quadriceps strength, knee joint position sense, and patient-reported function was completed for each athlete upon enrollment.

### Biomechanics testing

Kinematic data were recorded at 250 Hz using a 6-camera Motion Analysis system (raptor E with associated Cortex software). Kinetic data were collected at 1500 Hz using an AMTI force plate (AMTI, Watertown, Massachusetts) synchronized with the motion capture system. Retroreflective markers were placed on various anatomic landmarks of the pelvis and lower extremities in accordance with the Plug-in-Gait lower body marker set (right and left anterior superior iliac spines; right and left posterior superior iliac spines; lower lateral surface of the right and left thigh along the line between the hip and knee joint markers; right and left lateral epicondyle of the femur; lower lateral surface of the right and left tibia along the line between knee and ankle joint markers; right and left lateral malleolus; superior proximal end of the second metatarsal of the right and left foot; and posterior aspect of the Achilles tendon of the left and right leg at the same height as the second metatarsal marker). A static calibration trial was conducted with the athletes standing in the anatomical position. Following the static calibration trial, the athletes completed a standardized warm-up which involved various running and jumping tasks in order to become accustomed to the laboratory setting and the presence of the markers [23].

For the single-legged drop-landing task, participants started from a single-legged standing position on a 25 cm high platform placed next to the force plate. The athlete stood on the ACLR limb, jumped onto the force plate, landing on it with the same limb, and then jumped upward as high as possible. Each athlete was allowed to practice the landing task four times. Three trials were collected for each participant. The mean of these three landings was submitted to statistical analysis. No feedback was given during data collection. Kinematic and kinetic data from the single-leg landing trials were filtered using a 4th order, zero-lag, recursive Butterworth filter. A cutoff frequency of 15 Hz was used for the marker data and a cutoff frequency of 50 Hz was used for the force data. Three-dimensional joint angles were calculated for the trunk, hip and knee using an XYZ Cardan sequence, which resulted in joint angles corresponding with flexion/extension, adduction/abduction, and internal/external rotation. Joint angles reflected the orientation of the local coordinate system of the distal segment relative to the local coordinate system of the proximal segment. All kinetic variables were identified during the first 100 ms following initial contact with the force plate. Loading rates were calculated by dividing the peak vGRF by the time to peak force [24]. All kinetic variables were normalized to body mass (e.g., Nm/kg) or bodyweight (BW) as appropriate. All data processing was performed using custom MATLAB scripts (The MathWorks, Inc., Natick, MA, USA) [25] to extract peak angles for trunk, hip, and knee flexion, knee abduction, and knee internal rotation, peak anterior tibial shear force, peak knee extension and abduction moments, loading rate, and peak vGRF during the initial landing phase of the single-legged landing task. For each of these aforementioned dependent variables, the three-trial mean was calculated.

#### Knee joint position sense

Following biomechanics testing, athletes completed a passive repositioning testing protocol to assess the knee joint position sense of their ACL reconstructed limb. The testing protocol used in this study has been previously described in detail and demonstrates good test-retest reliability (ICC = 0.78) [22]. Briefly, athletes were seated upright in the isokinetic dynamometer (Biodex Medical System, Inc., Shirley, NY, USA) with their knee initially flexed to 90° and their eyes closed. Their knee was passively extended to 45° of knee flexion by the isokinetic dynamometer and held for 5 s before returning to the initial position (90° of flexion). We instructed athletes to try to remember the position of their knee during the 5-s hold. The knee was then passively moved into extension by the isokinetic dynamometer and athletes were asked to press a button when they thought their knee had returned to the target angle of 45° of flexion. The absolute difference between the knee angle at the time of the button press and the target angle (‘error’) was recorded. Each athlete completed 2 trials and the average error was calculated.

#### Patient-reported function

Athletes completed the Persian version of the International Knee Documentation Committee (IKDC) Subjective Knee Evaluation Form, which has been validated for use in Persian-speaking individuals after ACL injury [26]. The IKDC Subjective Knee Evaluation Form captures various aspects of knee-related function and is commonly used in athletes following ACL reconstruction [27]. Scores are expressed as a percentage, with 100% indicating full function and no symptoms. The minimal clinically important difference (MCID) for the IKDC has been reported to be 11.5 in athletes post-ACL reconstruction [28]. The MCID reflects the smallest amount of change in a measure that is perceived as meaningful.

We used the Tegner scale to capture the amount and types of physical activity that the athletes were participating in at the time of baseline testing. A higher Tegner score is indicative of a greater amount of activity and/or more demanding activities (ranges from 0 to 10) [27].

Pre and post-test assessments were conducted by the second athletic trainer, at baseline and after intervention. This investigator was blinded to group assignment.

#### Neuromuscular training program

Following completion of baseline testing, athletes in the experimental group participated in an 8-week progressive neuromuscular training program under the supervision of the third experienced athletic trainer. The program designed to improve lower extremity strength, control, power, balance, and landing technique. The program used in this study has been previously described and shown to improve hip strength and hop distance, and reduce high-risk landing mechanics, in uninjured athletes [29]. Athletes completed 3 sessions per week for weeks 1–6 and 2 sessions per week for weeks 7 and 8 (22 total sessions). Eight exercises were performed as part of the program: double-leg squats, walking lunges, single-leg squats, double-leg drop jumps, single-leg stance on an unstable surface, single-leg countermovement jumps, horizontal bounds, and single-leg standing long jumps. All exercises were performed with bodyweight resistance. Details regarding the exercises performed each week, as well as the sets and repetitions/time are provided in Table 1. The program components, duration, and frequency are consistent with current recommendations for primary ACL injury prevention programs [10]. Throughout training, the trainer provided athletes with standard instructions/cues regarding their technique in order to maximize the effectiveness of the program. The specific instructions for each exercise were based on those proposed by Benjaminse et al. (2015) [30]. and were intended to promote an external focus of attention, which has been shown to result in better performance and retention of learned movement patterns for a wide-range of movement tasks (vs. an internal focus) [31]. The specific instructions we provided are included in Table 2. Athletes in the control group continued to complete their routine activities which focused on sport-specific skills training over the same 8-week period but did not receive any formal neuromuscular training.

**Table 1:** Neuromuscular training program details

Exercise	Wk 1	Wk 2	Wk 3	Wk 4	Wk 5	Wk 6	Wk 7	Wk 8
Double-leg squats	3 × 6	3 × 6	–	–	–	–	–	–
Walking lunges	3 × 6	3 × 6	–	–	–	–	–	–
Single-leg squats	3 × 6	3 × 6	4 × 8	4 × 8	4 × 12	–	–	–
Double-leg drop jumps	–	–	3 × 6	4 × 10	4 × 12	–	–	–
Single-leg stance, unstable surface	–	–	3 x 30s	3 x 30s	4 x 30s	4 x 30s	3 x 30s	3 x 30s
Single-leg countermovement jumps	–	–	3 × 6	3 × 8	4 × 8	4 × 10	3 × 8	3 × 6
Horizontal bounds	–	–	–	–	–	4 × 8	5 × 10	3 × 8
Single-leg standing long jumps	–	–	–	–	–	4 × 8	5 × 8	3 × 8

^a^Sets and repetitions or time for each exercise across the 8-week period

^b^Wk = week

^c^Athletes given 30–60 s of rest between sets

**Table 2:** Instructions/cues provided to athletes in the experimental group during each exercise

Exercise	Instructions/Cues
Double-leg squat	While bending your knees, point your knees toward the cones and pretend you are going to sit on a chair while keeping a ball between your knees **Notes:** Cones positioned in line with neutral knee positions.
Walking lunge	While pretending you have a plank on your back, point your knee toward an imaginary point in front of you.
Single-leg squat	Stand on one leg and reach slowly toward the cone with your knee while bending your knee. **Notes:** Cone positioned in line with neutral knee position.
Double-leg drop jump	Jump down from the box, land on the markers on the floor, and point your toes and knees toward the cones. **Notes:** Cones positioned in line with neutral knee positions; 30 cm high box.
Single-leg stance, unstable surface	Keep the bar horizontal. **Notes**: Athlete held bar in front of them during exercise.
Single-leg countermovement jump	Jump as high as you can and touch the hanging ball. **Notes:** Ball included as overhead goal; height adjusted for each athlete.
Horizontal bound	Push against the ground as forcefully as possible.
Single-leg standing long jump	Try to jump past the line. **Notes:** Target line provided; distance adjusted for each athlete.

After the 8-week period, follow-up assessments of biomechanics, knee joint position sense, and patient reported function were completed for the athletes in both the experimental and control groups. The testing procedures and materials we used during this follow-up session were consistent with those utilized during baseline testing.

##### Statistical analysis

We used two-tailed independent t-tests to compare the age, mass, height, and body mass index (BMI) for the athletes in the experimental and control groups, and a Mann-Whitney U test to compare Tegner Activity Scale scores.

We used two-tailed independent t-tests to compare the groups’ baseline performance for each variable. We used two-way ANCOVA with a between factor of group (experimental, control) and a within factor of time (baseline, follow-up) to compare how the groups responded over the 8-week period. In the case of a group-by-time interaction effect, we conducted as post hoc comparisons to examine changes within the groups (follow-up vs. baseline). We used an alpha of 0.05 for all tests of statistical significance. We used SPSS software for statistical analysis (IBM Corp., Armonk, NY, USA). Cohen’s d effect size (ES) statistic was calculated by dividing the difference between the means by the standard deviation from the baseline time point. Effect sizes of 0.2, 0.5, and 0.8 were considered ‘small’, ‘moderate’, and ‘large’ [19].

## Results

There was no difference between the control and experimental groups in age (*P* = 0.87), mass (*P* = 0.91), height (*P* = 0.44), BMI (*P* = 0.67), or Tegner scores (*P* = 0.36) (Table 3). There were also no differences between the groups at baseline for any of the dependent variables of interest (*P* ≥ 0.100), which indicates that the groups were comparable with respect to biomechanics and function. Athletes in the experimental group participated in each scheduled training session (100% compliance). All athletes who completed baseline testing also returned for follow-up testing.

**Table 3:** Athlete demographics.

	Control^a^	Experimental^a^	***P***^c^
Age, years	27.2 ± 3.3	26.9 ± 4.1	.87
Mass, kg	70.1 ± 6.4	70.3 ± 4.7	.91
Height, m	1.7 ± 0.1	1.8 ± 0.1	.43
BMI^b^, kg/m^2^	23.2 ± 2.9	22.7 ± 1.7	.67
Tegner score	4.5 (1–8)	6 (3–8)	.36
Time since surgery (months)	7.5 ± 1.4	7.8 ± 1.7	0.96

^a^Mean ± SD or median (range) for Tegner score

^b^BMI = body mass index

^c^*p*-values (*P*) based on independent t-tests or Mann-Whitney U test

The ANCOVA analyses indicated that there were group-by-time interaction effects for the peak trunk flexion (*P* < 0.001), peak hip flexion (*P* < 0.001), peak knee flexion (*P* < 0.001), peak knee abduction (*P* < 0.001), peak knee internal rotation (*P* < .001), position sense errors (*P* < 0.001) peak vGRF (*P* < 0.001), loading rate (*P* < 0.001), peak anterior tibial shear force (*P* < 0.001), peak knee extension moment (*P* < 0.001), and peak knee abduction moment (*P* < 0.001). Post hoc comparisons indicated that the experimental group demonstrated increased peak trunk (*P* = 0.003), hip (*P* = 0.008) and knee flexion (*P* = 0.012) during landing following the intervention. Further, the experimental group decreased peak knee abduction (*P* = 0.018), peak knee internal rotation angles (*P =* 0.022), loading rate (*P* = 0.016), peak anterior tibial shear force (*P =* 0.018), peak knee extension moment (*P* = 0.022), and peak knee abduction moment (*P* = 0.014), and position sense errors (*P* = 0.001) as well as peak vGRF (*P* = 0.008). There were no changes for the control group (Table 4).

**Table 4:** Performance during the baseline and follow-up sessions for the control and experimental groups

	Control Group					Experimental Group					
	Baseline^a^	Follow-up^a^	% Δ ^b^	***P***^*c*^	Effect size	Baseline^a^	Follow-up^a^	% Δ ^b^	***P***^*c*^	Effect size	Between group differences (ANCOVA)
**Kinematics**											
Peak trunk Flexion (°)	27.2 ± 10.8	28.3 ± 10.7	↑4.01	0.259	0.050	23.2 ± 10.3	48.3 ± 10.8	↑108.59	<.001	0.76	0.003*
Peak hip Flexion (°)	38.6 ± 10.9	37.9 ± 12.5	↓1.86	0.639	0.205	22.3 ± 6.8	35.3 ± 5.1	↑58.37	<.001	0.73	0.008*
Peak knee flexion (°)	23.5 ± 9.1	27.3 ± 9.2	↑16.34	0.211	0.030	39.7 ± 12.54	56.8 ± 10.3	↑43.21	.01	0.60	0.012*
Peak knee abduction (°)	7.8 ± 1.1	7.6 ± 0.9	↓2.68	0.114	0.101	8.1 ± 1.18	5.7 ± 0.8	↓29.64	<.001	0.77	0.018*
Peak knee internal rotation (°)	14.4 ± 1.7	14.5 ± 1.9	↑0.27	0.870	0.001	15.9 ± 2.15	12.8 ± 1.2	↓19.86	<.001	0.76	0.022*
**Kinetics**											
Peak reaction force, N/BW	3.9 ± 1.2	3.8 ± 1.1	↓1.81	0.075	0.030	3.4 ± 1.19	2.2 ± 0.5	↓34.21	<.001	0.54	0.008*
Peak anterior Tibial shear force (BW)	0.8 ± 0.5	0.8 ± 0.7	0	0.085	0.027	0.8 ± 0.6	0.7 ± 0.5	↓12.5	0.01	0.66	0.018*
Peak knee extension moment (Nm/kg)	3.8 ± 1.1	3.9 ± 0.8	↑2.6	0.120	0.029	3.5 ± 1.3	2.7 ± 0.7	↓22.85	0.01	0.59	0.022*
Peak Knee Abduction Moment (Nm/kg)	1.5 ± 1.2	1.6 ± 1.1	↑6.66	0.073	0.032	1.6 ± 0.7	1.0 ± 0.8	↓37.5	<.001	0.64	0.014*
Loading rate (BW/S)	45.4 ± 10.7	46.9 ± 8.1	↑3.3	0.093	0.022	47.6 ± 6.9	34.1 ± 8.5	↓28.36	0.01	0.81	0.016*
Position sense errors (°)	6.7 ± 3.7	6.5 ± 2.9	↓2.96	0.684	0.030	5.8 ± 1.67	2.8 ± 1.1	↓51.90	.01	0.73	0.001*
IKDC scores (%)	67.3 ± 8.1	68.8 ± 11.5	↑2.2%	.55	0.075	65.6 ± 9.7	84.7 ± 1.8	↑29.1%	<.001	0.80	0.003*

^a^Mean ± standard deviation for each dependent variable of interest during the baseline and follow-up time points

^b^% Δ = percent change (follow-up relative to baseline); Iso = isometric, Con = concentric, Ecc = eccentric;

IKDC = International Knee Documentation Committee

^*c*^*p*-values (*P*) related to post hoc paired t-tests

*denoted significant differences (ANCOVA)

The ANCOVA analyses indicated that there were group-by-time interaction effects for the IKDC scores (*P* < 0.001). Post hoc comparisons indicated that the experimental group increased IKDC scores (*P* = 0.003) following training, while there was no change in IKDC scores for the control group (*P* = 0.550) (Table 4). Importantly, the increase in IKDC scores for the experimental group (19.1%) exceeded the MCID associated with the measure (11.5%) (Table 4).

## Discussion

This study aimed to examine the effects of a neuromuscular training program that emphasizes external focus of attention cuing on biomechanics, knee proprioception, and patient-reported function in athletes who had undergone ACL reconstruction and completed conventional post-operative rehabilitation. It is demonstrated that neuromuscular training programs using external focus of attention, such as the one used in our study, could promote improvements in landing biomechanics, proprioception, and patient-reported function in athletes with a history of ACL reconstruction.

The results of our study showed that neuromuscular training with external focus decreased loading rate and peak anterior tibial shear force. Considering that increased anterior tibial shear force is associated with increased ligament loading, this is a beneficial finding [32]. It is also reported that tibial shear force and consequently loading rate are associated with the quadriceps and hamstring muscles’ characteristics. Studies have shown that quadriceps force produces anterior tibial shear force and introduces stress and strain to the ACL with the knee near full extension [33, 34]. Conversely, the hamstrings provide posterior tibial shear force, subsequently reducing the force placed on the ACL [35]. Blackburn and colleagues (2013) also stated that peak anterior tibial shear force and loading on ACL are smaller in the individuals with higher hamstrings stiffness [36]. Quadriceps and hamstring muscle forces contribute to the net shear force at the tibiofemoral joint, and therefore have important implications for ACL injury during functional tasks such as jump landing [33]. In the neuromuscular group the patients were provided with strength exercises while receiving external focus instruction. Although muscle activation and strength were not measured in the present study, we postulate that the decrease in tibial shear force and loading rate could be the results of improved dynamic function of the quadriceps and hamstrings after 8-weeks of training.

Neuromuscular training also increased trunk, hip, and knee flexion and decreased knee abduction and internal rotation compared to control participants. Reduced hip and knee flexion and increased knee abduction and internal rotation may collectively increase the risk of ACL injury [29, 37]. That our intervention can reduce these hazardous joint positions is beneficial to the patient. Our findings are consistent with recent evidence suggesting that neuromuscular training with an externally directed focus of attention, may be beneficial for ACLR rehabilitation and prevention of ACLR injury [38].

Athletes who completed our neuromuscular training program demonstrated reductions in landing forces (Table 4). Previous studies that have investigated the effects of similar programs incorporating strength training, plyometric exercise, and movement re-training have also observed significant reductions in landing forces [39]. This is encouraging, as softer landings would likely reduce ACL loading [40]. Importantly, our participants accompanied this reduction in vGRF with reductions in knee extension and abduction moments. Reducing the knee extension moment is important to decreasing ACL injury risk. The internal knee extension moment is reflective of, among other factors, increased quadriceps muscle activity [41, 42]. leading to increase anterior tibial shear force and ACL loading, during landing. Increased knee abduction moments have been suggested to contribute to ACL injury risk [43–45]; therefore, reducing all of these hazardous loads through neuromuscular training can be beneficial.

In this study, neuromuscular training emphasizing an external focus of attention yielded a 51% improvement (from 5.8 at baseline to 2.8 at follow-up stage) in position sense errors. The large improvement in joint position sense suggests that neuromuscular training using an external focus of attention may be a necessary adjunct to standard post-operative rehabilitation. Previous neuromuscular training programs in patients after ACLR have demonstrated improvement (from 5.42 to 4.45 degrees) in joint position sense [46]. We believe the difference between our study and those previously conducted is due to methodological differences in the neuromuscular training approach. The previous studies used neuromuscular training with an internal focus of attention emphasis, whereas the present study relied on external focus of attention during neuromuscular training. So, given that external focus incorporated into neuromuscular training exercises can significantly mitigate defects in proprioception after ACLR, it is recommended to use neuromuscular training with an external focus of attention emphasis for these patients. In order to maximize the effects of a neuromuscular training program it may be critical for patients to perform exercises with proper technique by receiving feedback that promotes an external focus of attention from the clinician.

It is worth noting that there were differences in hip and knee flexion angles between groups at baseline. Specifically, control participants landed with more hip, but less knee flexion compared to the experimental group. This low knee flexion posture may suggest that participants in the control group were quadriceps dominant. Previous research from our lab has demonstrated similar landing positions (e.g., less than 30-degrees knee flexion) in females with established quadriceps dominance [43]. Further evidence in support of control participants being quadriceps dominant was the excessive landing contact noise noted by our investigators during testing.

From a physiological point of view, the improved joint position sense observed in this study and the characteristics of peripheral receptors can be connected; however, physiological responses of the proprioception and joint movement have not been investigated. Joint position sense improvement may be due to higher order central nervous system (CNS) adaptations to the peripheral signals from Iα muscle spindles and joint receptors at the slow velocities and type II or dynamic muscle spindles at the fast movement velocities [34].

In the present study, patients in the intervention group demonstrated a 17% improvement in IKDC scores at follow-up. Previous authors have reported that the MCID for the IKDC ranges from 6.3 to 16.7 during the first 6 and 12 months, respectively, post-surgery [47]. Therefore, it can be concluded that the exercises present in this study improved patient satisfaction with the injured knee. This may be because the exercises in this study are very close to the athlete’s daily movements and the athlete can keep in touch with the movements, thus improving his or her progress and feeling satisfied with their performance.

We believe that the results of our study provide valuable insight regarding the effects of neuromuscular training with an external focus of attention in athletes who have undergone ACL reconstruction; however, our study has limitations that should be considered. First, our study included a relatively homogenous sample of male athletes who had undergone hamstring autograft ACL reconstruction. As a result, we are unable to determine if our results generalize to female athletes and/or athletes who have undergone other types of ACL reconstruction procedures or had concomitant injuries. Previous studies have often used the uninjured limb as a reference standard for assessing recovery/function of the ACL reconstructed knee by creating limb symmetry indices. However, a limitation of this approach is that the uninvolved limb often becomes deconditioned during recovery, which can lead to an overestimation of the degree of function of the ACL reconstructed limb when the uninvolved limb is used as a reference standard [11]. Additionally, a group performing neuromuscular training with an emphasis on internal focus of attention was not included. Therefore, we are unable to determine if the changes observed in our participants were due to the neuromuscular training or the emphasis on external focus of attention instructions.

## Conclusions

Neuromuscular training with external focus of attention cueing improved landing biomechanics in patients after ACL reconstruction. The combination of neuromuscular training with external focus cueing beneficially mitigates second ACL injury risk factors and should be emphasized during and after traditional post-operative rehabilitation.

## Data Availability

The datasets used and/or analysed during the current study are publicly available from the corresponding author on reasonable request.

## Abbreviations

ACL: anterior cruciate ligament
IKDC: International Knee Documentation Committee questionnaire
RTS: return to sport
VGRF: vertical ground reaction force
RCT: randomized controlled trial
MCID: minimal clinically important difference
CNS: central nervous system
